# Perceptual learning modules in undergraduate dermatology teaching

**DOI:** 10.1111/ced.15201

**Published:** 2022-05-22

**Authors:** Alexander Salava, Viljami Salmela

**Affiliations:** ^1^ Departments of Dermatology, Allergology and Venereology University of Helsinki Helsinki Finland; ^2^ Department of Psychology and Logopedics University of Helsinki Helsinki Finland

## Abstract

**Background:**

Dermatological diagnosis depends highly on visual skills, and implicit nonanalytical proficiency plays a key role. To correctly diagnose skin diseases, the clinician needs visual skills, and intuitive recognition plays a key role.

**Aim:**

To investigate the effectiveness of digital perceptual learning modules (PLMs) in undergraduate teaching, and how these affect medical students' learning about skin diseases.

**Methods:**

This was a study performed in Finland, which enrolled 39 students of an undergraduate dermatology course. Online PLMs designed for dermatology, using different pictures of skin diseases were performed three times: before, during and at the end of the course. The modules provided four outcome measures: diagnostic accuracy (percentage of correct responses), a rating of confidence about the decision, fluency (response/decision time) and a list of features on which the decision was based.

**Results:**

As the number of PLMs and the course duration increased, there were also improvements in the four measures, with a significant increase in diagnostic accuracy [from 66% to 94%; *P* < 0.001; partial η^2^ (η^2^_p_) = 0.92], fluency (as measured by a decrease in response time (from 10 to 6 s; *P* < 0 0.001; η^2^_p_ = 0.69) and self‐perceived confidence (2.5 to 4.3; *P* < 0 0.001, η^2^_p_ = 0.86) with subsequent PLMs and course duration. There was a diversification of recognized features, an increase in pattern recognition, and better attention to localization and contextual association. Based on student feedback, the PLMs functioned well online, and enhanced motivation and learning.

**Conclusion:**

PLMs increased diagnostic accuracy, had a positive effect on learning outcomes and were easily integrated alongside clinical teaching. Considering the current era of digital technologies, we believe that there is potential for wider use of PLMs to improve visual skills and strengthen implicit learning in dermatology.

## Graphical abstract

We studied the use of perceptual learning modules (PLMs) in dermatology teaching. Digital PLMs improved the diagnostic accuracy of skin conditions and self‐perceived confidence in undergraduate students. There is considerable potential for a wider use of PLMs to improve visual skills and strengthen implicit learning in dermatology.

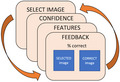

## Introduction

The diagnosis of skin conditions greatly depends on visual skills, which are essential in the practice of medicine.^1^ In addition to analytical knowledge, implicit nonanalytical proficiency plays a key role in comparing and categorizing lesions.^2^, ^3^ Important aspects include morphology, pattern recognition, distribution and contextual factors.^4^ Visual pattern recognition, discrimination and perceptual learning (PL) has been studied with psychophysical methods, e.g. discrimination thresholds and identification accuracies.^5^ There are also studies related to PL to improve implicit visual skills.^6^

In undergraduate education, visual skills are traditionally trained with bedside rounds and clinical pictures, but digital methods are increasingly being integrated.^4^, ^7^ There have been studies on the use of art in developing visual skills but learning with digital clinical images has been studied markedly less.^1^, ^8^ PL modules (PLMs) improve visual skills, but their outcomes in dermatology are unclear, and there is limited research on nonanalytical learning processes and intuitive recognition.^6^, ^9^

We investigated a novel PLM containing visual pattern recognition and feature selection combined with confidence rating and analytical feedback to study four outcome measures. In addition, we wanted to explore the settings and practicability of the digital learning environment regarding PLMs in dermatology education.

## Methods

### Students and perceptual learning modules

Medical students were offered anonymous participation in PLMs parallel to the dermatology course (fifth year of medical school, no prior teaching in dermatology or classification of skin lesions). There was no control group because PLMs were considered an integral teaching tool of the course, and we were mostly interested in the effects on learning during the course (within‐subjects design). The PLMs were online‐based and constructed using JavaScript and jsPsych^10^ software and run on a JATOS server.^11^ The PLMs contained clinical pictures selected by a single experienced dermatologist from the university's teaching pictures. The conditions comprised core curricular content (frequent skin‐related dermatoses) with special emphasis on relevance in primary care (Supplementary Data S1).^12^ The pictures were anonymized so that the patients were not identifiable and no additional patient data (e.g. history, sex, age) were provided.

Each trial of the PLM consisted of three displays. In the first display, four different images (diagnoses) were shown side‐by‐side with a question (Supplementary Figure S1). The questions were, for example: ‘Which of these is rosacea?’, then the participant needed to select the correct image to match the diagnosis. In total, 47 different diagnoses (tasks) were presented once in random order. In the second display, the image that the participant chose was shown again, and the participant was requested to select one or multiple visual features on which they had based their decision. A list of six features was shown (primary and secondary skin lesions, shape, location, context, e.g. age, sex, general impression and other features). In addition, the participant had to rate their decision confidence on a five‐point scale (very unconfident, quite unconfident, neutral, quite confident, very confident). This provided us with metacognitive knowledge on the task performance, which was expected to increase as function of learning. Finally, in the third display, the correct image was shown side‐by‐side with the chosen image as well as the cumulative percentage correct of all trials to provide immediate feedback and enhance learning motivation.

Each participant conducted two different modules three times: at the start, mid‐way through and at the end of the course. In total, each participant made 6 × 47 = 282 comparisons/decisions. On each task repetition, different images were used, and each image was shown only once. The three distractor images for each target image were chosen based on highest pixel‐wise correlation (increasing difficulty). In total, there were 1094 images, 282 target images and 846 distractors (6 × 47 × 3).

### Outcome measures

The PLMs provided four outcome measures: percentage of correct responses, confidence about the diagnosis, decision/response times and a list of features on which the decision was based. These were analysed by first averaging different tasks/diagnoses for each participant, and then comparing the measures across module repetitions. Participants filled out a questionnaire before and after the course (pre‐course and post‐course questionnaires, respectively) (Supplementary Data S2).

### Statistical analysis

The means of the correct responses, confidence ratings and decision times as well as the percentages of selected features were compared with separate two‐way repeated measures ANOVA [first factor: test time (three levels, pre‐, mid‐ and post‐course); second factor: repetition of PLM (two levels)]. The pre‐ and post‐course questionnaires were compared by paired samples *t*‐test. For effects sizes of different variables, we reported partial η^2^ (η^2^_p_) and Cohen *d* statistic. All *P* values of *post‐ho*c comparisons in ANOVAs were corrected for multiple comparisons. Statistical analyses were conducted with jamovi (https://www.jamovi.org) and R^13^ software, and *P* < 0.05 was considered statistically significant.

## Results

All 39 students from the course took part in the PLMs: 18 participants finished all 6 tests, 11 participants finished 3–5 tests, and 10 finished only 1–2 tests. Of the 39 students, only 25 conducted at least 1 test before, during and after the course, which may have been due to the shortness of the dermatology course (intensive, module‐type course of 3.5 weeks). Some students who participated in the PLMs did not want to participate in the study.

### Diagnostic accuracy

**Figure 1:**
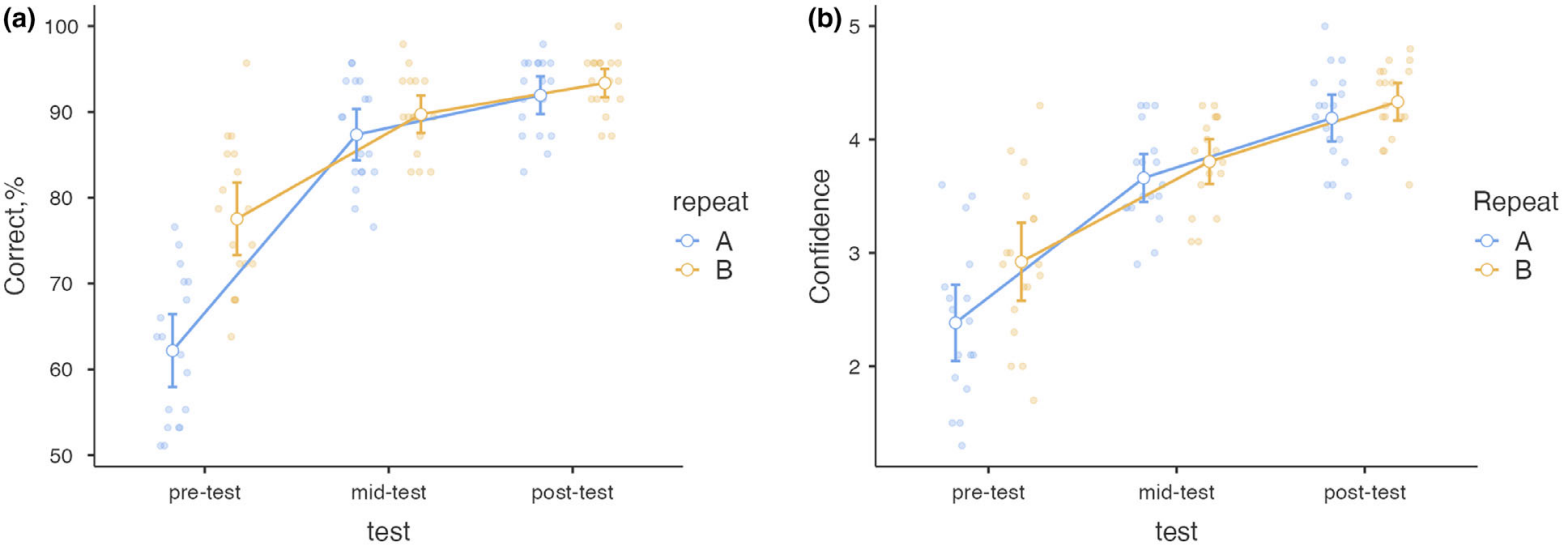
Correct responses (a) and confidence ratings (b) in two clustered pre‐, mid‐ and post‐tests. Repeat A indicates the first test, repeat B the second test.

The diagnostic accuracy was relatively high at baseline (mean ± SD 65.6 ± 9.95%) and increased progressively with subsequent PLMs and reached 93.8 ± 3.56% at course end (Fig. 1a).

The effects of course and PLMs were tested with repeated measures ANOVA, with test time (pre, mid, post) and repeat (A, B) as factors. The main effect of both test time (*F*_(2,34)_ = 207.6, *P* < 0.001, η^2^_p_ = 0.92) and repeating of PLMs (*F*_(1,17)_ = 37.1, *P* < 0 0.001, η^2^_p_ = 0.69), as well as their interaction (*F*_(2,34)_ = 18.2, *P* < 0.001, η^2^_p_ = 0.52) were statistically significant. All the *post‐hoc* comparisons were also significant (Table 1). The effect size was large for test time, but relatively small for repeat and interaction. The percentage of correct answers steadily improved during the course, but the effect of repeating PLMs was largest on the first occasion (Fig. 1a).

**Table 1:** *Post‐hoc* comparisons of correct responses, confidence ratings and decision/response times, as well as post‐course self‐evaluation of perceptual skills.

	Comparison	Difference	SE	df	*t*	*p*_Tukey_
Correct diagnosis	Pre vs. mid	−18.69	1.337	17	−13.98	< 0.001
	Pre vs. post	−22.80	1.228	17	−18.57	< 0.001
	Mid vs. post	−4.11	0.985	17	−4.17	0.002
	A vs. B	−6.38	1.05	17	−6.09	< 0.001
Confidence rate	Pre vs. mid	−1.081	0.0946	17	−11.43	< 0.001
	Pre vs. post	−1.608	0.1480	17	−10.87	< 0.001
	Mid vs. post	−0.528	0.0923	17	−5.72	< 0.001
	A vs. B	−0.276	0.0390	17	−7.07	< 0.001
Response time	Pre vs. mid	1.30	0.344	17	3.78	0.004
	Pre vs. post	2.49	0.274	17	9.09	< 0.001
	Mid vs. post	1.19	0.232	17	5.16	< 0.001
	A vs. B	1.55	0.187	17	8.27	< 0.001
		N	Mean	SD		
Improvement of visual skills		21	4.90	0.301		
PLMs improved visual skills		21	4.57	0.746		
Functionality of PLMs		21	4.24	0.889		
Meaningfulness of PLMs		21	4.43	0.598		
PLMs enhanced motivation		21	4.19	0.750		

df, degrees of freedom; *t*, *t*‐value of statistical test (ANOVA); PLM, perceptual learning module; *P*_Tukey_, *P* value corrected for multiple comparisons by Tukey test; SE, standard error.

### Confidence rating

The self‐perceived confidence rating increased similarly to diagnostic performance, although more gradually. Confidence increased from 2.5 pre‐course to 4.3 at course end (Fig. 1b).

The main effects of both test time (*F*_(2,34)_ = 102.47, *P* < 0 0.001, η^2^_p_ = 0.86) and repeating of PLMs (*F*_(1,17)_ = 49.97, *P* < 0 0.001, η^2^_p_ = 0.75), as well as their interaction (*F*_(2,34)_ = 3.95, *P* < 0.03, η^2^_p_ = 0.19) were all statistically significant. All the *post‐ho*c comparisons were also significant (Table 1). The effect size was large for test time, but very small for repeat and interaction. The confidence ratings steadily improved after each consecutive test (Fig. 1b).

### Fluency (decision time)

**Figure 2:**
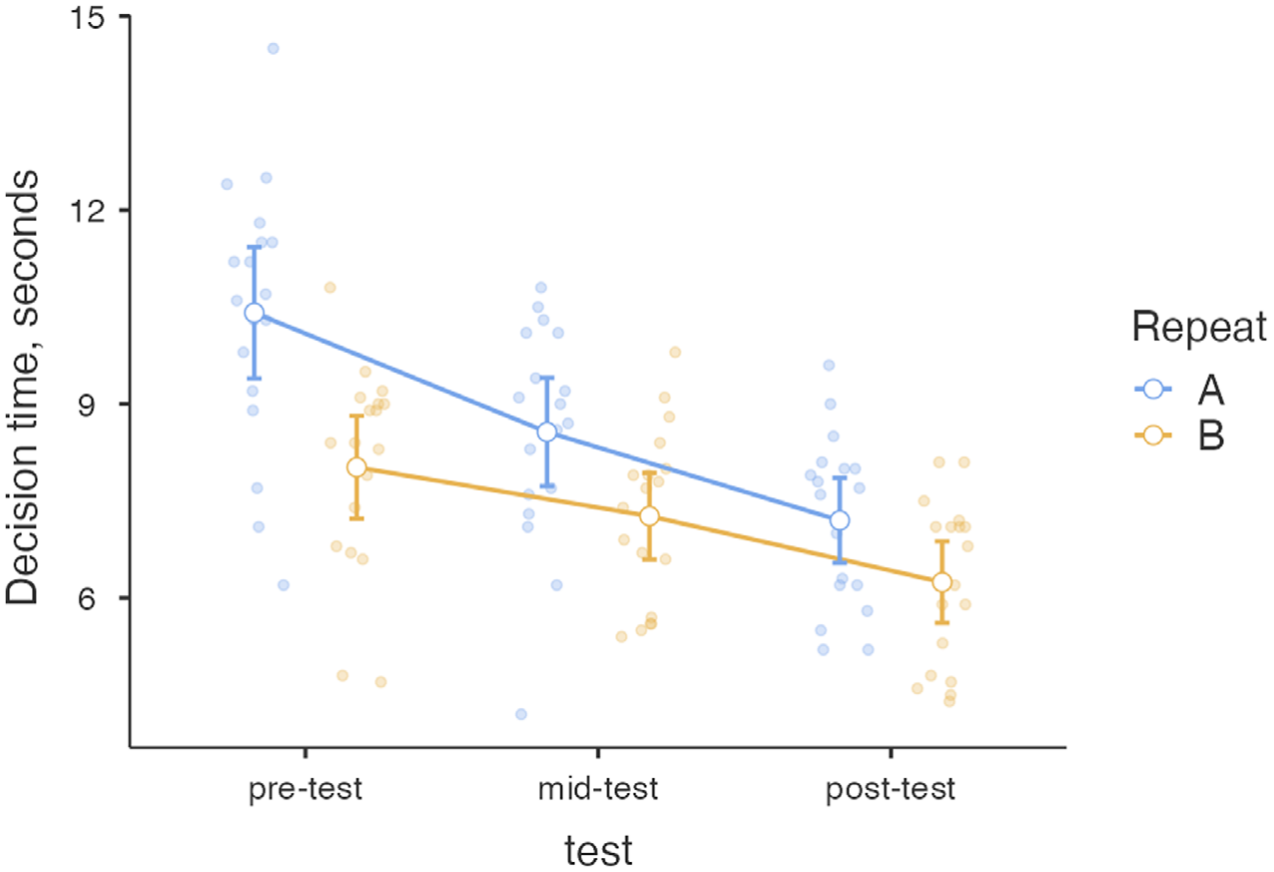
Decision times in two pre‐, mid‐ and post‐tests (repeated tests A and B).

Improvement in visual skills also produced a linear decrease in fluency, with decision times decreasing from 10 s pre‐course to 6 s at course end (Fig. 2).

The main effects of both test time (*F*_(2,34)_ = 37.75, *P* < 0 0.001, η^2^_p_ = 0.69) and repeating of PLMs (*F*_(1,17)_ = 68.45, *P* < 0.001, η^2^_p_ = 0.80) were statistically significant, but their interaction (*F*_(2,34)_ = 3.14, *P* < 0.06, η^2^_p_ = 0.16) was not. All the *post‐hoc* comparisons were also significant (Table 1). The effect size was medium for test time, but small for repeat.

### Frequencies of the recognized features

**Figure 3:**
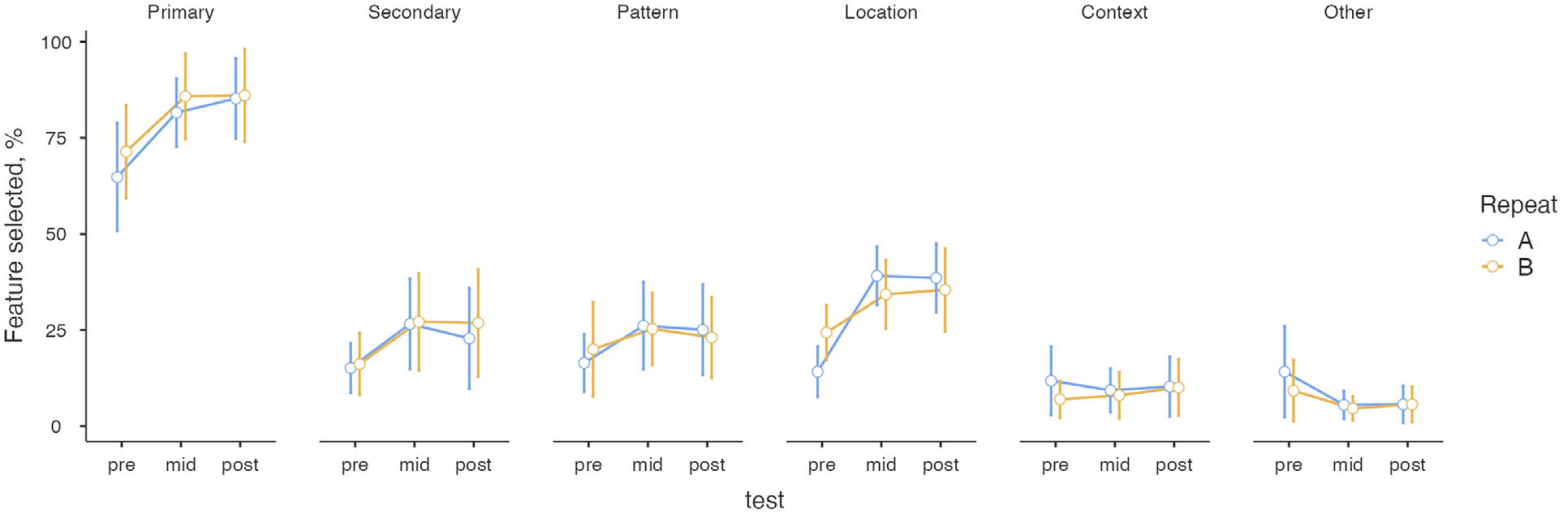
Percentages of selected features in two pre‐, mid‐ and post‐tests (repeated tests A and B). Feature groups: primary lesions, secondary lesions, pattern recognition, location of the lesions, context of the picture, other important features.

The selected amount of primary (*F*_(2,34)_ = 1.00, *P* < 0.001, η^2^_p_ = 0.37) and secondary (*F*_(2,34)_ = 5.24, *P* = 0 0.01, η^2^_p_ = 0.24) lesions, pattern recognition (*F*_(2,34)_ = 3.71, *P* < 0.04, η^2^_p_ = 0.18) and location (*F*_(2,34)_ = 42.17, *P* < 0.001, η^2^_p_ = 0.71) increased during the course duration (Fig. 3). The strongest effects were found for primary lesion and location. The feature ‘context’ remained relatively stable. The group of ‘other’ features decreased, but not statistically significantly.

During the study period, self‐perceived level of visual skills (*t*_(18)_ = 7.66, *P* < 0.001, *d* = 1.76) and proficiency in describing lesions (*t*_(18)_ = 9.86, *P* < 0.001, *d* = 2.26) increased significantly. Students' opinions about the role of visual skills in medicine were initially high, and underwent no significant changes (*t*_(18)_ = 90.57, *P* < 0 0.58, *d* = 0.13). Most students considered that their visual skills improved, and that PLMs helped to improve these skills (Table 1). Perceived practicality was high, and most students considered PLMs worthwhile and a good motivational aid.

## Discussion

The results of this study show that undergraduates enrolled in a dermatology course improved their diagnostic accuracy, fluency and self‐confidence when taking repeated digital PLMs.^14^ The students' performance was proportional to the sequential test number and was not equal in clustered paired tests, which indicates that the PLMs themselves affected performance. After successive PLMs, improvement plateaued, and the increase was relatively smaller when compared with previous tests but still visible until the final modules. In addition, the results show how feature detection and pattern recognition increased, and how the basis of the correct diagnosis expanded to include multiple features. The group of ‘other’ features consequently decreased, showing diversification, increase in pattern recognition, and attention to location and context. Thus, PLMs not only seem to facilitate diagnostic skills in dermatology but also widen the spectrum of recognized visual findings.^6^

Methods of PL have been developed for different settings and specialties, and there seems to be potential both in explicit and implicit dimensions of learning.^15^ Regarding dermatology, there are only a few studies about PL, even though dermatology is a predominantly visual speciality. Rimoin *et al*.^16^ studied adaptive PLMs used by medical students to identify skin lesion morphology, configuration and anatomical site; the results showed the usefulness of PLMs for developing clinical skills. Rourke *et al*.^17^ conducted a meta‐analysis on learning methods to identify skin lesions, and observed that the most effective methods involve high numbers of coordinated actions conducted at intervals, thus providing repeated practice. The observations are similar with our results, which show progressive performance improvement with intermittent PLMs.

The self‐perceived confidence rate (the metacognitive knowledge on the task performance) may increase faster with PL than with traditional methods.^6^, ^18^ One possible explanation might be the immediate feedback provided by the PLMs. In our study, students' confidence increased progressively with course duration and PLMs. The paired modules showed similar results, indicating that the actual time point of the PLM in the course may play an important role compared with accuracy.^9^

Evered^19^ observed that implicit training on paired cell images may be an effective method of visual learning in cytopathology, but only if the categorical borders between images are respected. In our study, only a single category corresponded to the correct diagnosis. We included an analytical feedback mechanism into the PLMs because, in addition to intuitive recognition, a systematic, explicit framework exists in dermatology and can be trained in parallel. Roads *et al*.^20^ investigated the easy‐to‐hard training advantage on clinical pictures of melanoma and benign skin tumours, and found that both easy‐to‐hard and hard‐to‐easy programmes were equally effective. We did not predict the clinical difficulty of our PLMs but based on the high baseline diagnostic accuracy it may be reasonable to increase difficulty by adding first display images and shortening response time.^14^, ^16^

Response time has been used in PLMs to modify task difficulty and strengthen nonanalytical learning.^6^, ^21^ We used a response time of 15 s, which has been described earlier in PL related to skin conditions.^2^, ^6^ Champagne *et al*.^22^ examined the effect of PLMs on the ability of medical residents to estimate left ventricular ejection fraction during echocardiography, and found significant improvements in time; however, the learning effect was not sustained at 6 months. We did not include a delayed post‐test measure for long‐term constancy but we plan to offer participants the same PLMs during sixth‐year courses (i.e. 1 year after the original PLMs) and thus investigate learning retention.^17^ It would also be interesting to study real‐life application of PLMs, e.g. for primary care doctors.

Based on student feedback, the PLMs were found to be practicable and easy to complete. Students considered that their visual skills had improved significantly and attributed this to the PLMs.

A possible limitation of our study was the parallel design with the dermatology course, which has an impact on the interpretation of the sole effect of PLMs. However, we were mostly interested in the learning during the course and how PLMs can be integrated into clinical education.^14^ Although the sample size was relatively small, the statistical power and effect size were considerable because of the number of images and repetitions.

## Conclusion

The present study shows how learning outcomes and self‐reported confidence may be enhanced with PLMs in teaching of dermatology. PLMs seem to function well in online settings, are easily integrated alongside clinical teaching and can be modified according to the needs of different specialties.^23^ Considering the current era of digital technologies, we believe that there is a considerable potential for a wider use of PLMs to improve visual skills and strengthen visual learning in dermatology.^24^

**What's already known about this topic?**

• Techniques of PL are used to enhance visual nonanalytical proficiency and improve learning outcomes.

**What does this study add?**

• Digital PLMs improve diagnostic accuracy of skin conditions and self‐perceived confidence in undergraduate students.
• There is considerable potential for wider use of PLMs to improve visual skills and strengthen implicit learning in dermatology.

## Funding

There were no funding resources that supported this work.

## Conflict of interest

The authors declare that they have no conflicts of interest.

## Ethics statement

The study was approved by the university's ethics review board (approval no. 35/2021). Students provided informed consent to participate.

## Data availability

The data that support the findings of this study are available from the corresponding author upon reasonable request. An example of the perceptual leaning module (without images) and instructions to construct perceptual learning modules with your own images can be found under the following link: https://www.mv.helsinki.fi/home/vsalmela/PLM/.

## Supporting information


**Data S1.** Appendix 1: list of dermatological entities (patient cases) used in perceptual learning modules.

**Data S2.** Appendix 2: pre‐test and post‐test questionnaires.

**Figure S1.** Structure and characteristics of the perceptual learning module in the e‐learning based environment.
