# Supplementary material for: Perceptual learning modules in undergraduate dermatology teaching
Source: Clin Exp Dermatol. 2022 May 22;47(12):2159–65. doi: 10.1111/ced.15201 (PMC10084265; doi:10.1111/ced.15201)
Supplement: Supplementary file 1 — Data S1. Appendix 1: list of dermatological entities (patient cases) used in perceptual learning modules. [file CED-47-2159-s003.docx]

**Appendix S1**

List of dermatological entities (patient cases) used in perceptual learning modules (alphabetical order).

The conditions comprised core curricular content (frequent skin-related dermatoses) with special emphasis on relevance in primary care.

1. Acne
2. Actinic keratosis
3. Allergic contact dermatitis
4. Angioedema
5. Atopic dermatitis
6. Basal cell carcinoma
7. Boil and furunculosis
8. Bullous pemhigoid
9. Candidal intertrigo
10. Dermatitis
11. Epidermal inclusion cyst
12. Erysipelas
13. Erythema multiforme
14. Erythema nodosum
15. Exanthema (drug or viral rash)
16. Folliculitis
17. Haemangioma
18. Herpes simplex
19. Herpes zoster
20. Impetigo contagiosa
21. Insect bites
22. Lichen simplex chronicus
23. Lichen planus
24. Lipoma
25. Lyme-borreliosis
26. Melanocytic nevus
27. Melanoma
28. Molluscum contagiosum
29. Nummular dermatitis
30. Palmoplantar pustulosis
31. Pediculosis capitis
32. Perioral dermatitis
33. Pityriasis (tinea) versicolor
34. Pityriasis rosea
35. Prurigo nodularis
36. Psoriasis
37. Purpura
38. Rosacea
39. Scabies
40. Seborrheic dermatitis
41. Seborrheic keratosis
42. Skin tag (Fibroma pendulans)
43. Squamous-cell carcinoma
44. Tinea (Ringworm)
45. Urticaria
46. Venous stasis dermatitis
47. Warts (Verruca vulgaris)
