# Supplementary material for: Perceptual learning modules in undergraduate dermatology teaching
Source: Clin Exp Dermatol. 2022 May 22;47(12):2159–65. doi: 10.1111/ced.15201 (PMC10084265; doi:10.1111/ced.15201)
Supplement: Supplementary file 2 — Data S2. Appendix 2: pre‐test and post‐test questionnaires. [file CED-47-2159-s002.docx]

**Appendix S2**

Participants filled out a questionnaire before (pre-test) and after (post-test) the course. The initial questionnaire contained the following questions (5-point Likert scale):

How good do you consider your visual perception skills?

Visual skills are important in clinical work.

How well can you describe cutaneous findings?

The questionnaire after course completion included the before mentioned questions and additionally:

My visual observation skills improved with the course.

PLMs helped improve my visual observation skills.

PLMs functioned well.

It was worth doing the PLMs.

PLMs motivated me to improve my visual skills.
