# Supplementary figures and images for: Perceptual learning modules in undergraduate dermatology teaching
Source: Clin Exp Dermatol. 2022 May 22;47(12):2159–65. doi: 10.1111/ced.15201 (PMC10084265; doi:10.1111/ced.15201)

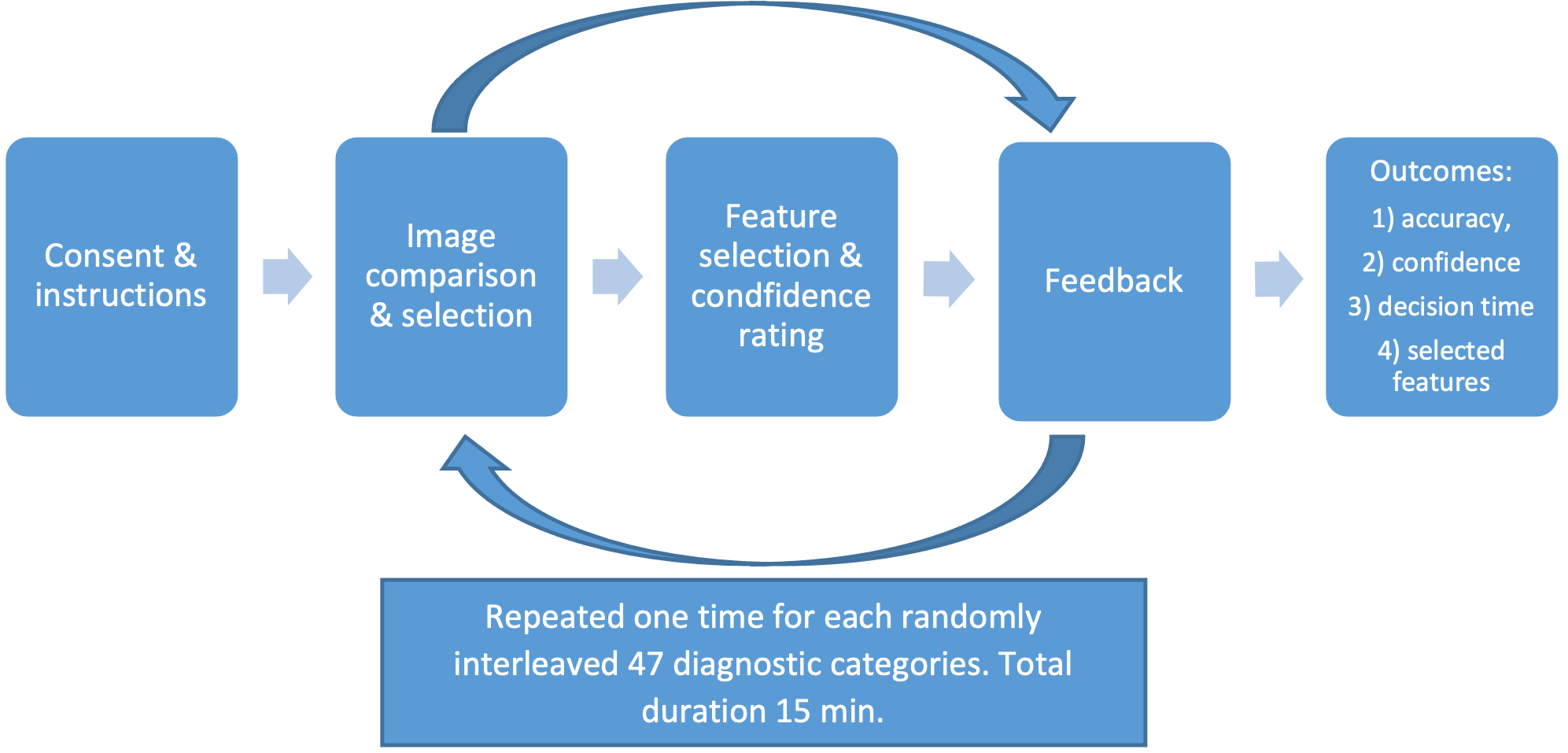

Supplement: Supplementary file 3 — Figure S1. Structure and characteristics of the perceptual learning module in the e‐learning based environment. [file CED-47-2159-s001.png]
